# The ratio of extracellular water to total body water serves as a potential predictor of diabetic peripheral neuropathy in patients

**DOI:** 10.3389/fendo.2025.1560902

**Published:** 2025-10-17

**Authors:** Lu Chen, Junli Zhang, Zhenghui Xu, Mengting Huang, Fei Hua, Yu Fu

**Affiliations:** ^1^ The Third Affiliated Hospital of Soochow University, Changzhou, China; ^2^ Department of Clinical Nutrition, The First People's Hospital of Changzhou, Changzhou, China; ^3^ Department of Endocrinology and Metabolism, The First People's Hospital of Changzhou, Changzhou, China; ^4^ Department of Geriatrics, the First Affiliated Hospital of Soochow University, Suzhou, China

**Keywords:** extracellular water/total body water ratio, type 2 diabetes mellitus, diabetic peripheral neuropathy, early predictive indicator, fluid balance, BIA

## Abstract

**Background:**

Diabetic peripheral neuropathy (DPN), a common chronic complication of type 2 diabetes mellitus (T2DM), lacks simple biomarkers for early monitoring. This study aimed to explore the association between the ratio of extracellular water to total body water (ECW/TBW) and DPN.

**Methods:**

A total of 707 T2DM patients recruited from the Third Affiliated Hospital of Soochow University were included in this cross-sectional study. Multivariate logistic regression analyses were performed to assess the association between the ECW/TBW ratio and DPN after adjusting. Receiver operating characteristic (ROC) curves were used to evaluate the predictive value of the ECW/TBW ratio for DPN.

**Results:**

The risk of DPN is related significantly with ECW/TBW ratio by multivariate logistic regression analyses, especially the ECW/TBW ratio of arms, trunk, and legs. And the ECW/TBW ratio can not be a indicator to predict the DPN rick of whose BMI is above 28kg/m². Besides, adding the ECW/TBW ratio to the baseline model gained a positive change in the integrated discrimination improvement and continuous net reclassification improvement. The area under the curve (AUC) of ECW/TBW (AUC:0.678) was higher than that of Neutrophil-to-Lymphocyte Ratio (NLR, AUC:0.620) and Platelet-to-Lymphocyte Ratio (PLR, AUC:0.568).

**Conclusions:**

The ratio of ECW/TBW exhibits a potential predictive capacity for DPN and better than NLR and PLR in T2DM patients with BMI <28 kg/m².

## 1 Introduction

Diabetes is primarily a chronic metabolic disorder related to lifestyle, affecting millions worldwide. Diabetic peripheral neuropathy (DPN) is one of its most common chronic complications (1), with a prevalence as high as 53% in China according to a multicenter study (2). DPN exerts a profound influence on the development of diabetic foot and non-traumatic diabetic amputation and will increases the risk of diabetic foot amputation without timely awareness and proper treatment (3, 4). Traditionally, DPN diagnosis relies on electromyography and clinical symptoms. However, by the time a DPN diagnosis is made, nerve damage has often progressed to an irreversible state (5, 6). Therefore, the investigation of indicators that can provide early warning signs and possess diagnostic value for DPN, especially those that are non-invasive and easily measurable, is of utmost importance.

Bioelectrical Impedance Analysis (BIA) is a simple, non-invasive tool for objectively assessing body composition, including fat, protein, minerals, and body water. In healthy individuals, total body water (TBW) accounts for approximately 60%-70% of body weight, further divided into intracellular water (ICW) and extracellular water (ECW) (7). The extracellular water-to-total body water ratio (ECW/TBW), a key parameter for evaluating cellular fluid balance, is closely associated with patients’ body composition statuses such as malnutrition, inflammation, and fluid retention conditions (e.g., ascites, pleural effusion, and peripheral edema). The normal ECW/TBW range is 0.360-0.390, with values exceeding 0.390 indicating edema. Measurable via BIA, the ECW/TBW ratio exceeding 0.400 indicates fluid overload in clinical practice (7, 8).

Fluid imbalance refers to disruptions in the body’s normal distribution, volume, or osmotic pressure of intracellular and extracellular fluid. It has been linked to poor clinical outcomes in patients with viral liver diseases, cancer, sarcopenia, and hemodialysis-dependent conditions (9–12). Fluid overload engages in complex crosstalk with inflammation, oxidative stress, and mitochondrial dysfunction which are three key pathological changes in DPN. Specifically, fluid overload triggers hypoxia, nutrient deficiency, and energy insufficiency, which in turn induce cascading reactions involving inflammation, oxidative stress, and mitochondrial dysfunction (13, 14). Early studies proposed that elevated sorbitol levels in Schwann cells induce osmotic overhydration and endoneurial edema in peripheral nerves of experimental diabetic models (15, 16). Jakobsen et al. (12) demonstrated significantly higher nerve water content in diabetic rats compared with controls, while Eaton’s cohort study established endoneurial edema as an early structural abnormality which are predating electrophysiological derangements, neurological deficits, and overt clinical neuropathy. A Singaporean cross-sectional analysis by Low et al. (17, 18) demonstrated that higher ECW/TBW ratios correlate with cognitive impairment in T2DM patients. Their subsequent prospective cohort study further revealed that excess extracellular volume independently predicts the progression of chronic kidney disease (CKD) in this population.

Growing evidences have established a robust association between fluid imbalance and diabetes mellitus (19). As a key indicator of fluid homeostasis, the ECW/TBW ratio has been applied in clinical assessments for diseases including colorectal cancer, hepatocellular carcinoma, cognitive impairment in type 2 diabetes, and diabetic kidney disease. However, whether ECW/TBW can serve as a novel biomarker for predicting DPN remains unclear. This study aims to investigate the relationship between ECW/TBW and DPN, specifically exploring its potential as an early predictor for DPN development.

## 2 Materials and methods

### 2.1 Study population

**Figure 1:**
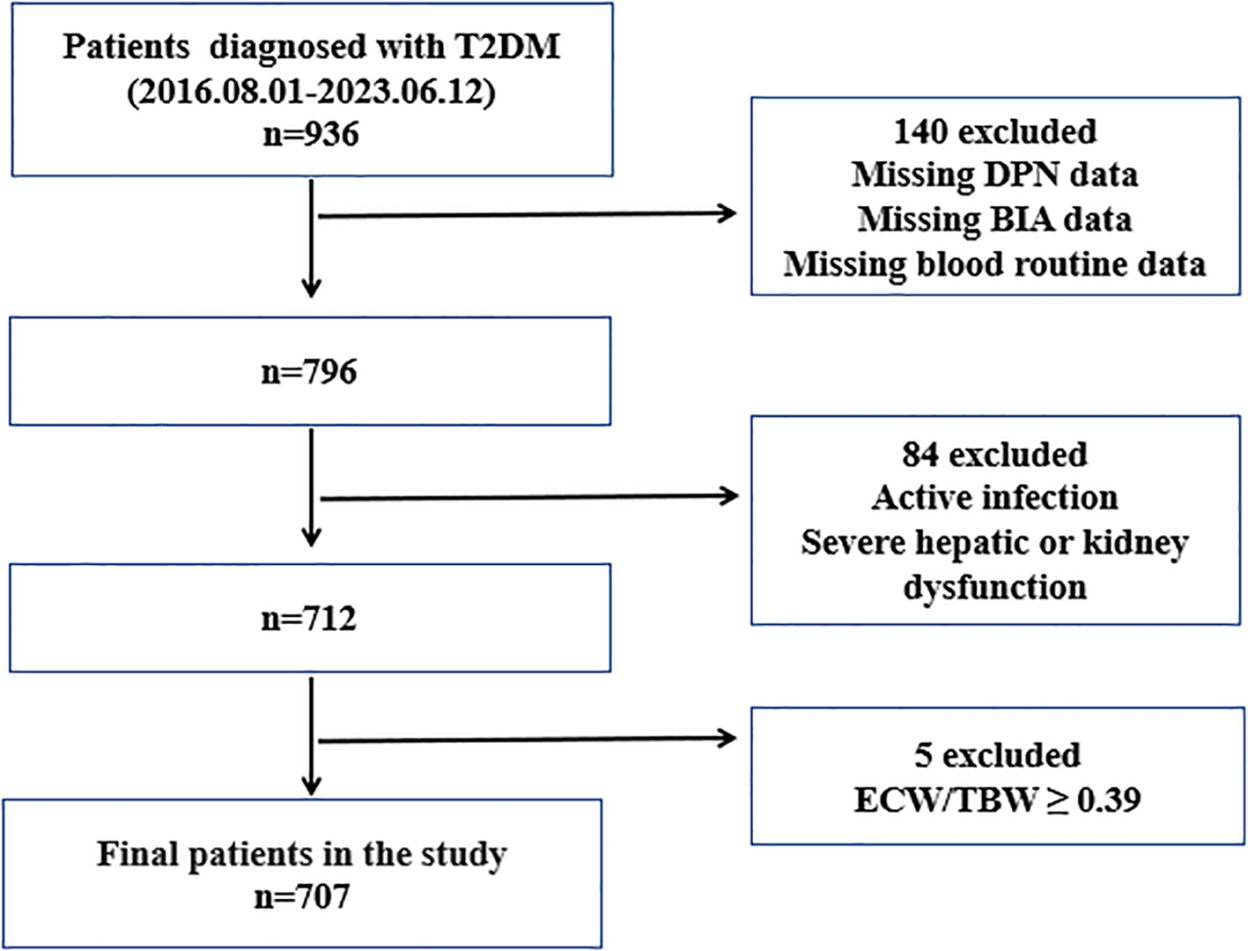
Flowchart of participant selection. T2DM, type 2 diabetes mellitus; DPN, diabetic peripheral europathy; BIA, bioelectric impedance analysis; ECW, extracellular water; TBW, total body water.

Between August 1, 2016, and June 12, 2023, 707 patients with type 2 diabetes mellitus (T2DM) were recruited from the Third Affiliated Hospital of Soochow University for this cross-sectional study (**Figure 1**). The diagnosis of T2DM and DPN was based on the Guideline for the Prevention and Treatment of Type 2 Diabetes Mellitus in China (2020 Edition) (20). Patients were excluded if they had type 1 diabetes mellitus, severe hepatic or renal dysfunction, severe infection, or malignant tumor; had missing data on electromyography, BIA, or blood routine tests; or had an ECW/TBW ratio ≥ 0.39. All eligible patients were categorized into a non-DPN group (n=395) and a DPN group (n=312). The diagnosis of DPN was based on a history of diabetes mellitus, presence of DPN-related symptoms or signs, and electromyographic evidence of reduced nerve conduction velocity. The workflow for participant selection is shown in **Figure 1**.

### 2.2 Data collection

Baseline characteristics of patients were obtained from electronic health records. Basic information included sex, age, duration of diabetes, smoking or alcohol history, systolic blood pressure (SBP), and diastolic blood pressure (DBP). Laboratory indicators included white blood cells (WBC), platelets, neutrophils, lymphocytes, monocytes, platelet-to-lymphocyte ratio (PLR), monocyte-to-lymphocyte ratio (MLR), neutrophil-to-lymphocyte ratio (NLR), alanine transaminase (ALT), aspartate aminotransferase (AST), total protein, albumin, fasting plasma glucose (FPG), fasting C-peptide (FCP), glycated hemoglobin A1c (HbA1c), total cholesterol (TC), triacylglycerol (TG), high-density lipoprotein (HDL), low-density lipoprotein (LDL), uric acid (UA), estimated glomerular filtration rate (eGFR), thyroid stimulating hormone (TSH), free triiodothyronine (FT3), and free thyroxine (FT4).

Homeostasis model assessment for insulin resistance (HOMA-IR) and homeostasis model assessment for islet beta-cell function (HOMA-islet) were calculated using the following formulas: The modified HOMA-IR was calculated as 1.5 + [FPG (mmol/L) × FCP (pmol/L)]/2800; the modified HOMA-islet was calculated as 0.27 × FCP (pmol/L)/[FPG (mmol/L) - 3.5] (21).

Body composition parameters were measured using BIA equipment (Inbody 770, Biospace, Seoul, Korea) by the same nutritionist (22). Prior to measurement, participants were instructed to remove personal metal items that might interfere with the procedure. They then stood on the equipment, held the electrodes in both hands, and awaited the results, which were obtained within a few minutes. Measurement outcomes included body mass index (BMI), total body water (TBW), intracellular water (ICW), and extracellular water (ECW).

### 2.3 Statistical analysis

All statistical analyses were performed using SPSS 26 and GraphPad Prism 9. Missing continuous variables were imputed via mean replacement, and categorical variables via mode replacement.

Normally distributed variables were presented as mean ± standard deviation, with group comparisons using Student’s t-test. Non-normally distributed variables were expressed as median and interquartile range, compared via the Mann-Whitney U test. Categorical variables were presented as frequencies (percentages), with group comparisons using the chi-square test.

Univariate and multivariate logistic regression were used to examine associations between ECW/TBW ratios (at different measurement sites) and DPN. Three models were constructed: Model 1 (no covariate adjustment), Model 2 (adjusted for sex and age), and Model 3 (adjusted for all covariates with statistically significant differences in univariate analysis). Subgroup analyses stratified by sex, age, and BMI were conducted to examine whether the association persisted.Receiver operating characteristic (ROC) curves were used to assess the ECW/TBW ratio’s predictive ability for DPN in T2DM patients. Net reclassification improvement (NRI) and integrated discrimination improvement (IDI) were calculated to evaluate its additional predictive value. Statistical significance was set at two-tailed P < 0.05.

## 3 Results

### 3.1 Baseline characteristics

A total of 707 T2DM patients were included in the final analysis. Of these, 312 were diagnosed with DPN and divided into the DPN group, based on the presence of definite DPN-related symptoms or signs plus electromyographic evidence. The remaining 395 patients, in contrast, were assigned to the non-DPN group.

Compared with non-DPN patients, those with DPN exhibited older age, higher levels of neutrophils, platelet-to-lymphocyte ratio (PLR), monocyte-to-lymphocyte ratio (MLR), neutrophil-to-lymphocyte ratio (NLR), fasting plasma glucose (FPG), total body water (TBW), intracellular water (ICW), extracellular water (ECW), and ECW/TBW ratio. In contrast, DPN patients had lower levels of lymphocytes, alanine transaminase (ALT), aspartate transaminase (AST), albumin, fasting C-peptide (FCP), HOMA-islet, estimated glomerular filtration rate (eGFR), and thyroid-stimulating hormone (TSH).

However, no statistically significant differences were observed between the two groups with respect to alcohol history, systolic blood pressure (SBP), diastolic blood pressure (DBP), white blood cell (WBC) counts, platelet counts, monocyte counts, total protein, glycated hemoglobin (HbA1c), HOMA-IR (insulin resistance index), total cholesterol (TC), triglycerides (TG), high-density lipoprotein (HDL), low-density lipoprotein (LDL), uric acid (UA), free triiodothyronine (FT3), free thyroxine (FT4), or body mass index (BMI) (**Table 1**).

**Table 1:** Comparison of baseline characteristics between the NDPN Group and the DPN Group.

Variable	NDPN Group (n=395)	DPN Group (n=312)	P value
Sex, male (%)	216.0 (54.7 %)	232.0 (74.4%)	<0.001***
Age (years)	55.0 (47.0, 63.0)	61.0 (52.3, 69.8)	<0.001***
Diabetes duration (months)	60.0 (6.0, 120.0)	108.0 (36.0, 180.0)	<0.001***
Smoking, n (%)	81.0 (20.5%)	105.0 (33.7%)	<0.001***
Drinking, n (%)	68.0 (17.2%)	70.0 (22.4%)	0.082
SBP (mmHg)	136.0 (123.0, 149.0)	137.0 (125.0, 149.0)	0.402
DBP (mmHg)	83.46 ± 11.34	83.21 ± 10.89	0.771
WBC (×10^9^/L)	6.20 (5.27, 7.27)	6.11 (5.25, 7.16)	0.757
Platelet (×10^9^/L)	207.00 (175.00, 239.00)	197.0 (171.00, 238.75)	0.182
Neutrophil (×10^9^/L)	3.44 (2.82, 4.33)	3.74 (2.99, 4.45)	0.035*
Lymphocyte (×10^9^/L)	2.00 (1.63, 2.44)	1.79 (1.46, 2.23)	<0.001***
Monocyte (×10^9^/L)	0.35 (0.29, 0.44)	0.37 (0.30, 0.44)	0.057
PLR	102.67 (81.72, 124.70 )	110.72 (88.67, 138.55)	0.002**
MLR	0.18 (0.14, 0.22)	0.21 (0.16, 0.26)	<0.001***
NLR	1.67 (1.32, 2.19)	2.01 (1.54, 2.69)	<0.001***
ALT (U/L)	19.30 (14.00, 32.00)	17.15 (12.00, 24.95)	<0.001***
AST (U/L)	18.80 (15.90, 25.90)	18.00 (14.73, 23.48)	0.017*
Total protein (g/L)	65.30 (62.60, 69.20)	64.75 (61.50, 69.20)	0.089
Albumin (g/L)	40.20 (38.50, 42.40)	39.40 (37.20, 41.80)	<0.001***
FPG (mmol/L)	8.20 (6.45, 10.83)	9.04 (7.02, 11.34)	0.015*
FCP (pmol/L)	613.80 (440.20, 800.00)	530.25 (374.43, 745.83)	0.001**
HbA1c (%)	9.10 (7.60, 10.90)	9.55 (7.90, 11.20)	0.100
HOMA-IR	3.33 (2.72, 4.12)	3.20 (2.56, 3.99)	0.101
HOMA-islet	34.53 (18.90, 59.01)	27.81 (14.47, 46.18)	<0.001***
TC (mmol/L)	4.45 (3.90, 5.20)	4.59 (3.86, 5.27)	0.890
TG (mmol/L)	1.74 (1.15, 2.53)	1.62 (1.12, 2.45)	0.536
HDL (mmol/L)	1.03 (0.89, 1.20)	1.03 (0.88, 1.22)	0.768
LDL (mmol/L)	2.58 (2.11, 3.17)	2.64 (2.12, 3.14)	0.984
UA (μmol/L)	309.60 (253.80, 376.50)	319.90 (259.68, 385.83)	0.200
eGFR (mL/min/1.73 min^2^)	103.38 (91.73, 115.49)	97.78 (84.19, 110.61)	<0.001***
TSH (uIU/mL)	1.98 (1.40, 2.82)	1.83 (1.26, 2.54)	0.020*
FT3 (pmol/L)	4.47 (4.05, 4.87)	4.47 (4.04, 4.83)	0.348
FT4 (pmol/L)	17.23 (15.39, 18.69)	17.19 (15.53, 18.22)	0.802
BMI (kg/m^2^)	24.20 (22.30, 26.70)	24.00 (22.00, 26.10)	0.183
TBW (kg)	34.70 (28.80, 40.40)	36.75 (32.33, 40.95)	0.002**
ICW (kg)	21.60 (17.70, 25.00)	22.50 (19.73, 25.18)	0.013*
ECW (kg)	13.30 (11.10, 15.40)	14.20 (12.60, 15.78)	<0.001***
ECW/TBW (%)	38.23 ± 0.68	38.69 ± 0.72	<0.001***
ECW/TBW (arms, %)	76.00 ± 0.92	76.39 ± 0.94	<0.001***
ECW/TBW (trunk, %)	38.23 ± 0.69	38.70 ± 0.74	<0.001***
ECW/TBW (legs, %)	76.57 ± 1.60	77.69 ± 1.69	<0.001***

*P <0.05, **P <0.01, ***P <0.001.

DPN, diabetic peripheral neuropathy; SBP, systolic blood pressure; DBP, diastolic blood pressure; WBC, white blood cells; PLR, platelet-to-lymphocyte ratio; MLR, monocyte-to-lymphocyte ratio; NLR, neutrophil-to lymphocyte ratio; ALT, alanine transaminase; AST, aspartate aminotransferase; FPG, fasting plasma glucose; FCP, fasting C-peptide; HbA1c, glycated hemoglobin A1c; HOMA-IR, homeostasis model assessment for insulin resistance; HOMA-islet, homeostasis model assessment for islet beta-cell function; TC, total cholesterol; TG, triglyceride; HDL, high-density lipoprotein; LDL, low-density lipoprotein; UA, uric acid; eGFR, estimated glomerular filtration rate; TSH, thyroid stimulating hormone; FT3, free triiodothyronine; FT4, free thyroxine; BMI, body mass index; TBW, total body water; ICW, intracellular water; ECW, extracellular water.

### 3.2 Correlation between ECW/TBW ratio and other indicators

According to spearman correlation analysis, the ratio of ECW/TBW was positively correlated with age, diabetes duration, SBP, PLR, MLR, NLR, and HDL. In contrast, it was inversely associated with sex, smoking status, alcohol consumption, DBP, WBC, ALT, AST, total protein, albumin, FPG, FCP, HOMA-IR, TC, TG, LDL, UA, eGFR, FT3, FT4,BMI,TBW, ICW, and ECW (**Supplementary Table S1**).

### 3.3 Binary logistic regression analysis to determine the relationship between ECW/TBW ratio and DPN

Binary univariate and multivariate logistic regression analyses were conducted to determine the predictors for DPN in the overall study population (**Table 2**). The result of univariate analysis indicated that sex, age, diabetes duration, smoking, lymphocyte, PLR, MLR, NLR, ALT, AST, total protein, albumin, FPG, FCP, eGFR, BMI, TBW, ICW, and ECW were associated with DPN. We excluded TBW and ECW from multivariate analysis owing to high collinearity with the ECW/TBW ratio. The result of multivariate analysis suggested the independent risk factors for DPN were sex, diabetes duration, FPG, and ICW. Additionally, univariate and multivariate analyses were performed to determine the correlation of ECW/TBW ratio at body different measured sites with DPN (**Table 3**). The association between ECW/TBW ratio and DPN was significant in all three regression models: Model 1 no variables for adjustment (OR = 2.580, 95% confidence interval: 2.041- 3.263, P<0.001); Model 2 adjusted for sex and age (OR = 3.301, 95% confidence interval:2.403 - 4.535, P<0.001); Model 3 adjusted for sex, age, diabetes duration, smoking, lymphocyte, PLR, MLR, NLR, ALT, AST, total protein, albumin, FPG, FCP, eGFR, BMI and ICW (OR = 3.122, 95% confidence interval: 2.202- 4.424, P<0.001). After adjusting for other covariates, it was found that T2DM patients were 2.122 times more likely to develop DPN for every unit increase in the ECW/TBW ratio (P < 0.001). ECW/TBW ratios of the arms, trunk, and legs was significantly correlated with the risk of DPN in three adjusted models (P < 0.05).

**Table 2:** Univariate and multivariate logistic regression between candidate covariates and DPN.

Variables	Univariate logistic regression		Multivariate logistic regression	
	OR (95% CI)	*P* value	OR (95% CI)	*P* value
Sex	2.403 (1.741, 3.317)	<0.001***	2.582 (1.299, 5.132)	0.007**
Age	1.044 (1.031, 1.058)	<0.001***	1.017 (0.994, 1.040)	0.146
Diabetes duration	1.005 (1.004, 1.007)	<0.001***	1.003 (1.000, 1.005)	0.017*
Smoking	1.966 (1.401, 2.759)	<0.001***	1.363 (0.883, 2.105)	0.162
Drinking	1.391 (0.958, 2.019)	0.083		
SBP	1.004 (0.995, 1.012)	0.380		
DBP	0.998 (0.985, 1.011)	0.770		
WBC	0.985 (0.891, 1.087)	0.759		
Platelet	0.998 (0.995, 1.001)	0.159		
Neutrophil	1.104 (0.974, 1.252)	0.121		
Lymphocyte	0.558 (0.431, 0.723)	<0.001***	0.918 (0.611, 1.379)	0.681
Monocyte	2.289 (0.730, 7.175)	0.155		
PLR	1.006 (1.002, 1.010)	0.001**	1.002 (0.996, 1.009)	0.440
MLR	252.075 (29.686, 2140.457)	<0.001***	2.414 (0.325, 17.908)	0.389
NLR	1.418 (1.203, 1.672)	<0.001***	1.041 (0.798, 1.359)	0.767
ALT	0.978 (0.969, 0.988)	<0.001***	0.991 (0.969, 1.014)	0.447
AST	0.977 (0.963, 0.992)	0.002**	1.014 (0.983, 1.047)	0.376
Total protein	0.973 (0.949, 0.998)	0.033*	1.025 (0.993, 1.058)	0.123
Albumin	0.924 (0.885, 0.964)	<0.001***	0.981 (0.931, 1.033)	0.458
FPG	1.043 (1.004, 1.084)	0.029*	1.081 (1.031, 1.133)	0.001**
FCP	0.999 (0.999, 1.000)	0.004**	1.000 (0.999, 1.000)	0.279
HbA1c	0.996 (0.983, 1.009)	0.547		
HOMA-IR	0.950 (0.854, 1.057)	0.349		
HOMA-islet	1.000 (0.999, 1.001)	0.832		
TC	0.993 (0.879, 1.121)	0.908		
TG	0.975 (0.927, 1.026)	0.330		
HDL	1.020 (0.662, 1.572)	0.928		
LDL	0.993 (0.838, 1.176)	0.934		
UA	1.001 (0.999, 1.002)	0.279		
eGFR	0.982 (0.975, 0.990)	<0.001***	0.989 (0.978, 1.000)	0.055
TSH	0.912 (0.811, 1.025)	0.124		
FT3	0.861 (0.690, 1.074)	0.185		
FT4	1.001 (0.972, 1.031)	0.951		
BMI	0.956 (0.917, 0.997)	0.036*	0.950 (0.883, 1.023)	0.177
TBW	1.034 (1.011, 1.057)	0.004**		
ICW	1.042 (1.006, 1.079)	0.022*	1.144 (1.046, 1.252)	0.003**
ECW	1.128 (1.062, 1.199)	<0.001***		

TBW and ECW were excluded from multivariate analysis owing to high collinearity with the ECW/TBW ratio. *P <0.05, **P <0.01, ***P <0.001.

DPN, diabetic peripheral neuropathy; SBP, systolic blood pressure; DBP, diastolic blood pressure; WBC, white blood cells; PLR, platelet-to-lymphocyte ratio; MLR, monocyte-to-lymphocyte ratio; NLR, neutrophil-to lymphocyte ratio; ALT, alanine transaminase; AST, aspartate aminotransferase; FPG, fasting plasma glucose; FCP, fasting C-peptide; HbA1c, glycated hemoglobin A1c; HOMA-IR, homeostasis model assessment for insulin resistance; HOMA-islet, homeostasis model assessment for islet beta-cell function; TC, total cholesterol; TG, triglyceride; HDL, high-density lipoprotein; LDL, low-density lipoprotein; UA, uric acid; eGFR, estimated glomerular filtration rate; TSH, thyroid stimulating hormone; FT3, free triiodothyronine; FT4, free thyroxine; BMI, body mass index; TBW, total body water; ICW, intracellular water; ECW, extracellular water.

**Table 3:** Correlation between ECW/TBW ratio at body different measured sites and DPN.

Variable	Model 1		Model 2		Model 3	
	OR (95% CI)	P value	OR (95% CI)	P value	OR (95% CI)	P value
ECW/TBW (%)	2.580 (2.041, 3.263)	<0.001***	3.301 (2.403, 4.535)	<0.001***	3.122 (2.202, 4.424)	<0.001***
ECW/TBW (arms, %)	1.586 (1.341, 1.875)	<0.001***	1.510 (1.248, 1.828)	<0.001***	1.447 (1.171, 1.788)	0.001**
ECW/TBW (trunk, %)	2.479 (1.974, 3.114)	<0.001***	3.393 (2.468, 4.665)	<0.001***	3.227 (2.275, 4.577)	<0.001***
ECW/TBW (legs, %)	1.518 (1.373, 1.678)	<0.001***	1.632 (1.430, 1.862)	<0.001***	1.611 (1.393, 1.863)	<0.001***

Model 1: unadjusted. Model 2: adjusted for sex and age. Model 3: adjusted for sex, age, diabetes duration, smoking, lymphocyte, PLR, MLR, NLR, ALT, AST, total protein, albumin, FPG, FCP, eGFR, BMI and ICW. **P <0.01, ***P <0.001.

ECW, extracellular water; TBW, total body water; DPN, diabetic peripheral neuropathy; PLR, platelet-to-lymphocyte ratio; MLR, monocyte-to-lymphocyte ratio; NLR, neutrophil-to lymphocyte ratio; ALT, alanine transaminase; AST, aspartate aminotransferase; FPG, fasting plasma glucose; FCP, fasting C-peptide; eGFR, estimated glomerular filtration rate; BMI, body mass index; ICW, intracellular water.

### 3.4 Subgroup analysis of the ECW/TBW ratio and DPN relationship

**Figure 2:**
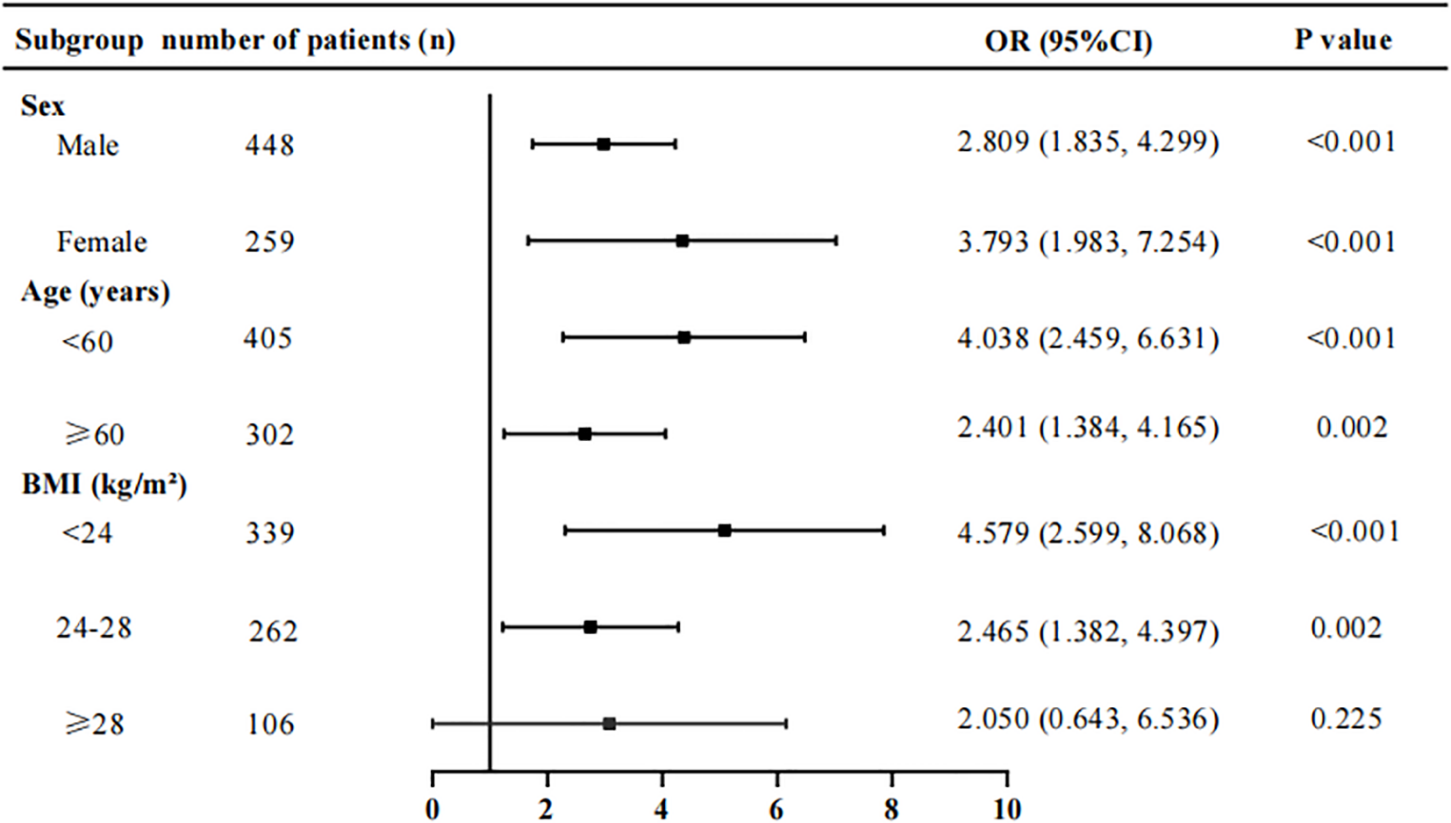
Stratification subgroup analysis on the association between ECW/TBW ratio and the risk of DPN. Adjusted for variables in Model 3. ECW, extracellular water; TBW, total body water; DPN, diabetic peripheral neuropathy; BMI, body mass index.

Subgroup analyses revealed that the ECW/TBW ratio was not associated with the risk of diabetic peripheral neuropathy (DPN) in patients with a BMI ≥ 28 kg/m², following adjustment for covariates as specified in Model 3 (**Figure 2**).

### 3.5 Diagnostic performance of the ECW/TBW ratio for DPN

**Figure 3:**
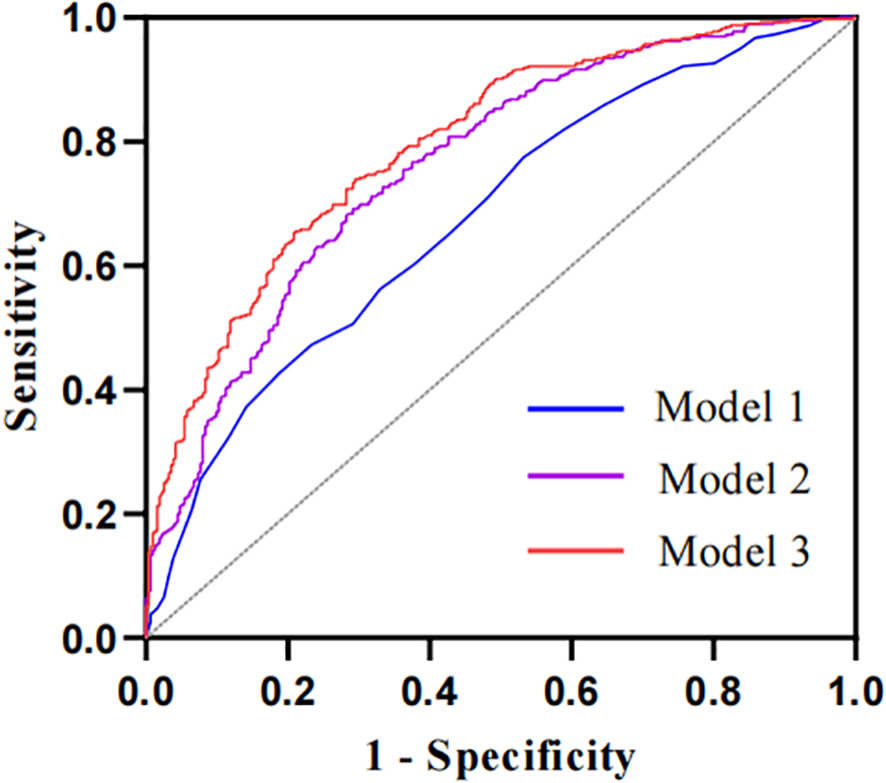
The ROC curves of the ECW/TBW ratio adjusted for different variables to predict DPN. The AUC of Model 1 is 0.678 (95% CI: 0.638-0.717) with no adjustment for covariates; the AUC of Model 2 is 0.762 (95% CI: 0.727-0.797) adjusted for baseline model; the AUC of the Model 3 is 0.796 (95% CI: 0.764-0.829) adjusted for ECW/TBW ratio in addition to the variables in baseline model. Baseline model includes sex, age, diabetes duration, smoking, lymphocyte, PLR, MLR, NLR, ALT, AST, total protein, albumin, FPG, FCP, eGFR, BMI and ICW.

The ROC curve analysis was applied to assess the diagnostic performance of the ECW/TBW ratio for DPN (**Figure 3**). The area under the curve (AUC) of the ECW/TBW ratio for predicting DPN was 0.678 (95% CI: 0.638-0.717) in Model 1 (unadjusted) and 0.762 (95% CI: 0.727-0.797) in Model 2 (adjusted for variables). After adding the ECW/TBW ratio to Model 2, the AUC increased to 0.796 (95% CI: 0.764-0.829). To assess the impact of the ECW/TBW ratio on the predictive ability of DPN occurrence, reclassification analyses were conducted. The results showed that the inclusion of the ECW/TBW ratio in the baseline model significantly increased the categorical NRI (0.138, P<0.001) and continuous NRI (0.427, P<0.001), as well as the IDI (0.054, P<0.001) (**Supplementary Table S2**). These findings indicated that the ECW/TBW ratio improved the ability to predict the presence of DPN.

### 3.6 Predictive ability of the ECW/TBW ratio, NLR, and PLR for DPN

**Figure 4:**
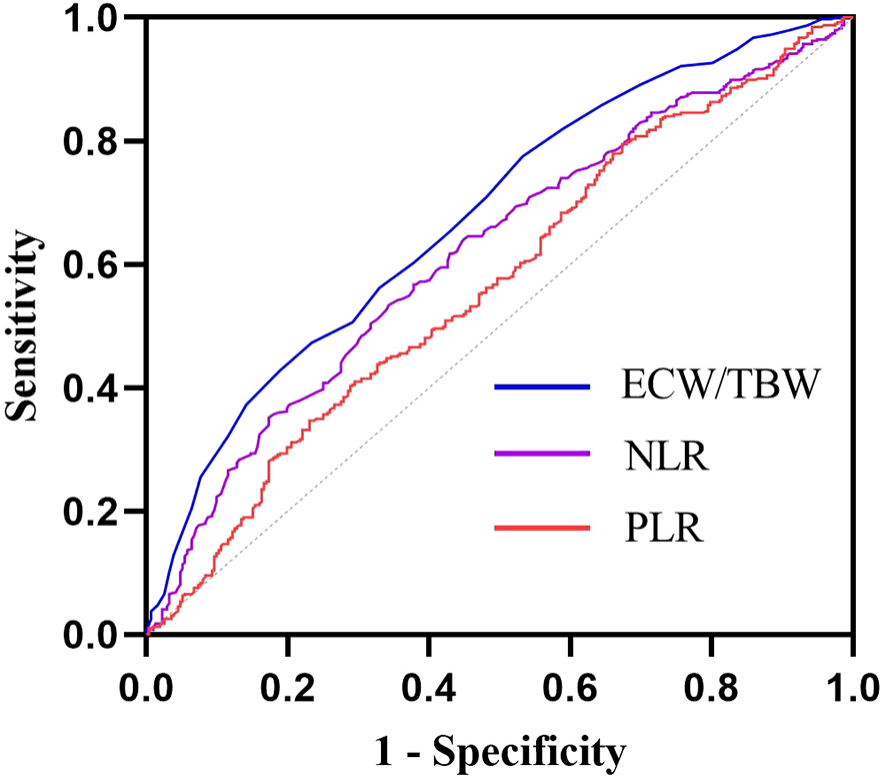
ROC curves of ECW/TBW, NLR and PLR for predicting DPN. The AUC of ECW/TBW for predicting DPN was 0.678 (95% CI: 0.638-0.717); The AUC of NLR for predicting DPN was 0.620 (95% CI: 0.579-0.661); The AUC of PLR for predicting DPN was 0.568 (95% CI: 0.526 0.611);.

To further evaluate the ability of the ECW/TBW ratio, NLR, and PLR to predict diabetic peripheral neuropathy (DPN) in patients with diabetes, receiver operating characteristic (ROC) curves were used. The area under the curve (AUC) for the ECW/TBW ratio was 0.678 (sensitivity = 46.8%, specificity = 77.5%), which was higher than those for NLR (AUC = 0.620) and PLR (AUC = 0.568) (**Table 4**, **Figure 4**). Additionally, we calculated the maximum Youden index and derived the optimal cutoff value of 0.388 for the ECW/TBW ratio to predict DPN.

**Table 4:** ROC analysis of ECW/TBW, NLR and PLR for predicting DPN.

Variables	AUC	95% Cl	Youden’s index	Sensibility	Specificity	P value
ECW/TBW (%)	0.678	0.638-0.717	38.75	46.8%	77.5%	<0.0001
NLR	0.620	0.579-0.661	0.196	65.7%	53.9%	<0.0001
PLR	0.568	0.526-0.611	0.122	32.7%	79.5%	0.0019

## 4 Discussion

This cross-sectional study demonstrated that higher ECW/TBW ratios were significantly associated with an increased risk of DPN. Notably, we found that ECW/TBW ratios in the arms, trunk, and legs each exhibited independent correlations with DPN risk. Results from the ROC curve analysis further validated the ECW/TBW ratio as a robust predictor for DPN occurrence in individuals with T2DM. A retrospective case-control study by Yang et al. demonstrated that patients with type 1 diabetes mellitus (T1DM) and DPN exhibited a significantly higher ECW/TBW ratio (0.3969 ± 0.0097) compared to those without DPN (0.3886 ± 0.0086; p < 0.001) (23 **).** While our research and Yang et al.’s study have reached similar conclusions, the key distinction lies in the patient populations we focused on. Specifically, our work focuses primarily on patients with type 2 diabetes mellitus (T2DM), whereas Yang et al.’s study was conducted in patients with type 1 diabetes mellitus (T1DM). Based on our study results and Yang’s, the ECW/TBW ratio emerges as a promising biomarker for early DPN detection and prevention, with demonstrated potential for clinical application in risk stratification and intervention planning. In addition, we confirmed that BIA is highly feasible for routine clinical care—particularly in T2DM management—owing to its non-invasiveness, speed, low cost, and indicative role in assessing DPN.

The underlying etiology of DPN remains incompletely elucidated, but it is widely accepted to involve a pathophysiological cascade of metabolic abnormalities, oxidative stress, and inflammation (24). Among these pathophysiological alterations, hyperglycemia, inflammatory responses, and oxidative stress are widely recognized as cardinal drivers of DPN development and progression.

Hyperglycemia has been shown to initiate inflammatory cascades and compromise Na+/K+ ATPase channel activity by activating key biochemical pathways, including the polyol, advanced glycation end products (AGEs), and protein kinase C (PKC) pathways. These pathways collectively culminate in neuronal and Schwann cell (SCs) damage (5). Additionally, hyperglycemia-induced PKC overactivation impairs Na+/K+ ATPase function and neurovascular perfusion by promoting vasoconstriction, further exacerbating neuroischemia (25). Our study establishes a significant association between elevated ECW/TBW ratios and increased DPN risk; however, the precise mechanistic underpinnings remain incompletely understood. Buscemi et al. postulate that diabetic hyperglycemia induces a mild osmotic gradient, driving water efflux from the intracellular to extracellular compartment and elevating the ECW/ICW ratio (26). This osmotic stress triggers a cascade of cellular derangements, including intracellular dehydration, cytoskeletal remodeling, and impaired enzymatic activity within the nucleus, mitochondria, and cytosol (27, 28). Prolonged osmotic imbalance culminates in cumulative damage, activating apoptotic pathways and inducing cell death (27). These findings implicate hyperglycemic osmolarity stress as a potential mediator linking elevated ECW/TBW ratios to DPN pathogenesis. Given these insights, future risk assessment models and preventive strategies for DPN should explicitly account for hyperglycemia-induced osmotic perturbations.

Inflammation and oxidative stress remain pivotal pathophysiological mechanisms in the pathogenesis of DPN (28, 29). Emerging evidence has firmly established that serum markers of inflammatory and oxidative stress responses are intimately associated with DPN progression (30, 31). Specifically, hyperglycemia, dyslipidemia, and insulin resistance synergistically activate the protein kinase C (PKC), polyol, hexosamine, advanced glycation end products (AGEs)/receptor for AGEs (RAGE) signaling cascades, which collectively induce inflammation, oxidative stress, mitochondrial dysfunction, and culminate in neuronal apoptosis (30, 32).

A seminal study by Park et al. demonstrated that extracellular fluid excess (ECF) potently exacerbates the risk of coronary artery calcification in chronic kidney disease (CKD) patients (33). The investigators proposed dual mechanistic pathways: First, ECF elicits endothelial release of angiotensin II, which activates angiotensin II type 1 receptor, thereby augmenting superoxide anion production and diminishing nitric oxide bioavailability (34). Second, ECF induces phenotypic transdifferentiation of vascular endothelial and smooth muscle cells, promoting oxidative stress-mediated vascular calcification (35, 36). Concomitantly, excess ECF has been linked to robust upregulation of inflammatory biomarkers, including tumor necrosis factor-α (TNF-α), interleukin-6 (IL-6), C-reactive protein (CRP), and macrophage infiltration (37–40). Moh et al. further demonstrated that elevated neutrophil-to-lymphocyte ratio (NLR) correlates with progressive renal function decline in type 2 diabetes mellitus (T2DM) patients, a phenomenon partially attributed to dysregulated fluid homeostasis (19).

From a mechanistic perspective, inflammatory states compromise endothelial barrier integrity, promoting interstitial fluid extravasation into the extracellular space and elevating the extracellular water to total body water ratio (ECW/TBW). Mitsides et al. proposed that in hemodialysis-dependent CKD, extracellular fluid imbalance exhibits a positive correlation with endothelial dysfunction markers (e.g., vascular cell adhesion molecule 1 [VCAM-1] and matrix metalloproteinase-1 [MMP-1]), underscoring a bidirectional link between overhydration and endothelial injury (38). Platelet-to-lymphocyte ratio (PLR), monocyte-to-lymphocyte ratio (MLR), and NLR serve as validated surrogates of vascular inflammation (41, 42). Our Spearman analysis revealed significant positive correlations between ECW/TBW and PLR, MLR, NLR, suggesting that ECW/TBW may potentiate DPN risk via inflammatory or oxidative stress-mediated pathways. However, large-cohort prospective studies are essential to validate this mechanistic association.

Our findings unequivocally identified sex, diabetes duration, and fasting plasma glucose (FPG) as independent risk determinants for diabetic peripheral neuropathy (DPN). Prolonged diabetes duration correlates with cumulative exposure to pathogenic factors, thereby escalating the incidence of complications. The male gender association with DPN aligns with prior epidemiological evidence (43, 44). Notably, this study unveiled intracellular water (ICW) as a novel independent risk marker for DPN—an observation of particular significance, as both intracellular fluid overload and depletion exert deleterious effects on somatic cells, including neuronal populations.

Supporting the “cell swelling theory,” emerging evidence demonstrates that cell volume acts as a dynamic metabolic sensor modulating cellular homeostasis (45, 46). *In vivo* models reveal that cellular swelling promotes anabolic pathways (e.g., glycogen synthesis), suppresses proteolytic activity, whereas cell shrinkage exacerbates catabolic processes and protein degradation (46, 47). These mechanistic insights underscore the critical need for maintaining fluid homeostasis in type 2 diabetic patients.

Total body fluid volume is known to be modulated by age, gender, and body habitus (48). Accordingly, we conducted subgroup analyses stratified by sex, age, and body mass index (BMI). Results demonstrated that the extracellular water to total body water ratio (ECW/TBW) was significantly associated with DPN risk across all subgroups, except for the BMI ≥28 kg/m² stratum. The absence of this association in the obese subgroup may be attributed to the limited sample size of type 2 diabetic patients. Larger-cohort studies are warranted to validate these findings.

Accumulating evidence indicates that neutrophil-to-lymphocyte ratio (NLR) and platelet-to-lymphocyte ratio (PLR) are associated with diabetic peripheral neuropathy (DPN), demonstrating predictive utility for DPN risk. As a pivotal marker of fluid homeostasis, the ECW/TBW has been applied in clinical assessments for various conditions, including colorectal cancer, hepatocellular carcinoma, cognitive impairment in type 2 diabetes, and diabetic kidney disease. Notably, our study revealed that the ECW/TBW ratio exhibited robust predictive capacity for DPN risk, as evidenced by a receiver operating characteristic (ROC) curve with an area under the curve (AUC) of 0.678—significantly higher than that of NLR (AUC: 0.620) and PLR (AUC: 0.568). Our findings Our results were highly consistent with Bo Lou’s (49)and Siying Liu’s (50). NLR emerged as noteworthy risk indicators associated with the manifestation of DPN in patients with type 2 diabetes and AUC of NLR was 0.661 according to Bo Lou’s data. And in Siying Liu’s study (50),AUC of NLR was 0.619 for diagnosing DPN in patients with T2DM.

This investigation entailed several critical limitations. First, the cross-sectional design inherently precluded causal inference between ECW/TBW ratio and DPN, as temporal sequence could not be established. Second, selection biases—including admission rate bias and prevalence-incidence bias—inevitably compromised the external validity of results, representing a fundamental constraint. Third, we failed to account for dynamic fluctuations in hydration status (e.g., diuretic use, dietary sodium intake, fluid retention disorders), which are known to substantially confound ECW/TBW measurements. Fourth, the small sample size inherently limited statistical power, necessitating interpretation of findings as preliminary.Specifically, we aim to acquire large-sample, multicenter datasets to validate the prognostic value of the ECW/TBW ratio in predicting DPN and to explore potential mechanisms underlying this association through prospective cohort studies in future.

Consequently, large-scale prospective cohort studies with longitudinal monitoring of hydration parameters are essential to validate these associations and establish causal mechanisms.

## 5 Conclusion

The ECW/TBW ratio proves to be a higher predictive capacity of DPN than NLR and PLR, highlighting its potential as a novel diagnostic indicator and be a potential indicator to predict the risk of DPN in T2DM patients whose BMI<28kg/m².

## Data Availability

The raw data supporting the conclusions of this article will be made available by the authors, without undue reservation.

## Supplementary material

Supplementary Table 1 The association between ECW/TBW ratio and other indicators. *P <0.05, **P <0.01, ***P <0.001. SBP, systolic blood pressure; DBP, diastolic blood pressure; WBC, white blood cells; PLR, platelet-to-lymphocyte ratio; MLR, monocyte-to-lymphocyte ratio; NLR, neutrophil-to lymphocyte ratio; ALT, alanine transaminase; AST, aspartate aminotransferase; FPG, fasting plasma glucose; FCP, fasting C-peptide; HbA1c, glycated hemoglobin A1c; HOMA-IR, homeostasis model assessment for insulin resistance; HOMA-islet, homeostasis model assessment for islet beta-cell function; TC, total cholesterol; TG, triglyceride; HDL, high-density lipoprotein; LDL, low-density lipoprotein; UA, uric acid; eGFR, estimated glomerular filtration rate; TSH, thyroid stimulating hormone; FT3, free triiodothyronine; FT4, free thyroxine; BMI, body mass index; TBW, total body water; ICW, intracellular water; ECW, extracellular water.

Supplementary Table 2 NRI and IDI for the incremental predictive values of ECW/TBW ratio. Baseline model includes sex, age, diabetes duration, smoking, lymphocyte, PLR, MLR, NLR, ALT, AST, total protein, albumin, FPG, FCP, eGFR, BMI and ICW. ***P <0.001. DPN, diabetic peripheral neuropathy; AUC, area under the curve; NRI, net reclassification index; IDI, integrated discrimination improvement; ECW, extracellular water; TBW, total body water.
