# Supplementary material for: The ratio of extracellular water to total body water serves as a potential predictor of diabetic peripheral neuropathy in patients
Source: Front Endocrinol (Lausanne). 2025 Oct 17;16:1560902. doi: 10.3389/fendo.2025.1560902 (PMC12575126; doi:10.3389/fendo.2025.1560902)
Supplement: Supplementary Table 1 — The association between ECW/TBW ratio and other indicators. *P <0.05, **P <0.01, ***P <0.001. SBP, systolic blood pressure; DBP, diastolic blood pressure; WBC, white blood cells; PLR, platelet-to-lymphocyte ratio; MLR, monocyte-to-lymphocyte ratio; NLR, neutrophil-to lymphocyte ratio; ALT, alanine transaminase; AST, aspartate aminotransferase; FPG, fasting plasma glucose; FCP, fasting C-peptide; HbA1c, glycated hemoglobin A1c; HOMA-IR, homeostasis model assessment for insulin resistance; HOMA-islet, homeostasis model assessment for islet beta-cell function; TC, total cholesterol; TG, triglyceride; HDL, high-density lipoprotein; LDL, low-density lipoprotein; UA, uric acid; eGFR, estimated glomerular filtration rate; TSH, thyroid stimulating hormone; FT3, free triiodothyronine; FT4, free thyroxine; BMI, body mass index; TBW, total body water; ICW, intracellular water; ECW, extracellular water. [file Table1.pdf]

Table S1 The association between ECW/TBW ratio and other indicators.

Variable	r	P
Sex	-0.316	<0.001***
Age	0.571	<0.001***
Diabetes duration	0.347	<0.001***
Smoking	-0.098	0.009**
Drinking	-0.108	0.004**
SBP	0.105	0.005**
DBP	-0.182	<0.001***
WBC	-0.099	0.008**
Platelet	-0.054	0.152
Neutrophil	-0.004	0.911
Lymphocyte	-0.235	<0.001***
Monocyte	-0.042	0.266
PLR	0.169	<0.001***
MLR	0.167	<0.001***
NLR	0.173	<0.001***
ALT	-0.379	<0.001***
AST	-0.248	<0.001***
Total protein	-0.231	<0.001***
Albumin	-0.407	<0.001***
FPG	-0.076	0.043*

Table S1 (continued)

**Variable**	**r**	**P**
FCP	-0.187	<0.001***
HbA1c	0.045	0.228
HOMA-IR	-0.187	<0.001***
HOMA-islet	-0.054	0.151
TC	-0.136	<0.001***
TG	-0.264	<0.001***
HDL	0.217	<0.001***
LDL	-0.101	0.007**
UA	-0.165	<0.001***
eGFR	-0.379	<0.001***
TSH	-0.012	0.744
FT3	-0.327	<0.001***
FT4	-0.091	0.016*
BMI	-0.328	<0.001***
TBW	-0.401	<0.001***
ICW	-0.449	<0.001***
ECW	-0.314	<0.001***

Note: * P <0.05, ** P <0.01, *** P <0.001.

Abbreviations: SBP, systolic blood pressure; DBP, diastolic blood pressure; WBC, white blood cells; PLR, platelet-to-lymphocyte ratio; MLR, monocyte-to-lymphocyte ratio; NLR, neutrophil-to lymphocyte ratio; ALT, alanine transaminase; AST, aspartate aminotransferase; FPG, fasting plasma glucose; FCP, fasting C-peptide; HbA1c, glycated hemoglobin A1c; HOMA-IR, homeostasis model assessment for insulin resistance; HOMA-islet, homeostasis model assessment for islet beta-cell function;

TC, total cholesterol; TG, triglyceride; HDL, high-density lipoprotein; LDL, low-density lipoprotein; UA, uric acid; eGFR, estimated glomerular filtration rate; TSH, thyroid stimulating hormone; FT3, free triiodothyronine; FT4, free thyroxine; BMI, body mass index; TBW, total body water; ICW, intracellular water; ECW, extracellular water.

Table S2 NRI and IDI for the incremental predictive values of ECW/TBW ratio.

	Presence of DPN	
	**Values**	**P value**
AUC (95% CI)		
Baseline model	0.762 (0.727-0.797)	
Baseline model + ECW/TBW ratio	0.796 (0.764-0.829)	
NRI (95% CI)		
Categorical	0.138 (0.075, 0.202)	0.001***
Continuous	0.427 (0.282-0.572)	0.001***
IDI (95% CI)	0.054 (0.037, 0.071) <	0.001***

Notes: Baseline model includes sex, age, diabetes duration, smoking, lymphocyte, PLR, MLR, NLR, ALT, AST, total protein, albumin, FPG, FCP, eGFR, BMI and ICW.

*** $P < 0.001$.

Abbreviations: DPN, diabetic peripheral neuropathy; AUC, area under the curve; NRI, net reclassification index; IDI, integrated discrimination improvement; ECW, extracellular water; TBW, total body water.
